# Immunomodulation and Reduction of Thromboembolic Risk in Hospitalized COVID-19 Patients: Systematic Review and Meta-Analysis of Randomized Trials

**DOI:** 10.3390/jcm10225366

**Published:** 2021-11-18

**Authors:** Dimitrios Sagris, Matilda Florentin, Panagiotis Tasoudis, Eleni Korompoki, Nikolaos Gatselis, Evangelos J. Giamarellos-Bourboulis, Haralampos Milionis, James Douketis, Alex C. Spyropoulos, George Dalekos, George Ntaios

**Affiliations:** 1 Department of Medicine and Research Laboratory of Internal Medicine, National Expertise Center of Greece in Autoimmune Liver Diseases, General University Hospital of Larissa, 413 34 Larissa, Greece; disagris@uth.gr (D.S.); tasoudis@gmail.com (P.T.); gatsel@me.com (N.G.); georgedalekos@gmail.com (G.D.); 2 Department of Internal Medicine, School of Medicine, University of Ioannina, 455 00 Ioannina, Greece; matildaflorentin@yahoo.com (M.F.); hmilioni@uoi.gr (H.M.); 3 Department of Clinical Therapeutics, Alexandra Hospital, University of Athens, 115 28 Athens, Greece; e.korompoki@imperial.ac.uk; 4 Division of Brain Sciences, Department of Stroke Medicine, Imperial College London, London SW7 2AZ, UK; 5 4th Department of Internal Medicine, Medical School, National and Kapodistrian University of Athens, 124 62 Athens, Greece; egiamarel@med.uoa.gr; 6 Department of Medicine, McMaster University, 1200 Main Street West, Hamilton, ON L8N 3Z5, Canada; jdouket@mcmaster.ca; 7 Department of Medicine, The Donald and Barbara Zucker School of Medicine at Hofstra/Northwell, Hempstead, NY 11549, USA; aspyropoul@northwell.edu; 8 The Institute for Health Innovations and Outcomes Research—The Feinstein Institutes for Medical Research, Manhasset, NY 11030, USA; 9 Anticoagulation and Clinical Thrombosis Services Northwell Health at Lenox Hill Hospital, New York, NY 10305, USA; 10 Department of Obstetrics and Gynecology, I.M. Sechenov First Moscow State Medical University, 119435 Moscow, Russia

**Keywords:** COVID-19, thromboembolism, tocilizumab, anakinra, hydroxycholoroquine, immunomodulation

## Abstract

Background: We aimed to investigate the potential beneficial effect of immunomodulation therapy on the thromboembolic risk in hospitalized COVID-19 patients. Methods: We searched PubMed and Scopus for randomized trials reporting the outcomes of venous thromboembolism (VTE), ischemic stroke or systemic embolism, myocardial infarction, any thromboembolic event, and all-cause mortality in COVID-19 patients treated with immunomodulatory agents. Odds ratios (OR) and 95% confidence intervals (CI) were calculated using the Mantel–Haenszel random effects method. Results: Among 8499 patients hospitalized with COVID-19, 4638 were treated with an immunomodulatory agent, 3861—with usual care only. Among the patients prescribed immunomodulatory agents, there were 1.77 VTEs per 100 patient-months compared to 2.30 among those treated with usual care (OR: 0.84, 95% CI: 0.61–1.16; I^2^: 0%). Among the patients who received an interleukin 6 (IL-6) antagonist, VTEs were reported in 12 among the 1075 patients compared to 20 among the 848 receiving the usual care (OR: 0.52, 95% CI: 0.22–1.20; I^2^: 6%). Immunomodulators as an add-on to usual care did not reduce the risk of stroke or systemic embolism (OR: 1.10, 95% CI: 0.50–2.40; I^2^: 0%) or of myocardial infarction (OR: 1.06, 95% CI: 0.47–2.39; I^2^: 0%) and there was a nonsignificant reduction in any thromboembolic event (OR: 0.86, 95% CI: 0.65–1.14; I^2^: 0%). Conclusions: We did not identify a statistically significant effect of immunomodulation on prevention of thromboembolic events in COVID-19. However, given the large effect estimate for VTE prevention, especially in the patients treated with IL-6 antagonists, we cannot exclude a potential effect of immunomodulation.

## 1. Introduction

Soon after the emergence of the SARS-CoV-2 pandemic and its associated COVID-19 disease, a high prevalence of thrombotic events, mostly consisting of venous thromboembolism (VTE), were observed in patients hospitalized with COVID-19. Meta-analyses of observational studies identified VTE prevalence ranging from 23.9% up to 40.3% in the patients who had undergone ultrasound screening [1,2]. Such thrombotic events occurred mainly in patients with severe disease, although they were also observed in mildly symptomatic or asymptomatic patients. It was postulated that thrombotic complications associated with COVID-19 were attributable, at least in part, to immune mechanisms that led to a hypercoagulable state [3,4,5].

Several proinflammatory cytokines, such as tumor necrosis factor-α (TNF-α), interleukin (IL) 1β and chemotactic cytokines (e.g., IL-8 and macrophage chemoattractant protein-1 (MCP-1)) are upregulated during COVID-19, leading to a sustained increase in IL-6 levels [6,7,8,9,10]. The latter seems to play a major role in the maintenance of the virus-driven inflammatory process. Alongside this inflammatory process, a prothrombotic effect was postulated through multiple mechanisms that include endothelial inflammation, destabilization of atherosclerotic plaques, release of the von Willebrand factor, and upregulation of coagulation and complement pathways [11]. These pathogenic mechanisms could lead to the formation of microthrombi in various vascular beds and, eventually, the development of clinically overt venous and arterial thrombosis.

Several immunomodulatory agents targeting IL-6 and IL-1 blockade were proposed as potential therapeutic options for severe COVID-19 to inhibit the proinflammatory effect and its consequences on pulmonary and other organ function [12,13,14,15,16,17,18,19,20,21]. Our group recently showed in the SAVE study that early soluble urokinase plasminogen activator receptor (suPAR)-guided anakinra use decreased the severe respiratory failure but did not affect the thromboembolic risk [16]. Recent meta-analyses of randomized and observational studies exploring the effect of anakinra and tocilizumab in patients with COVID-19 showed a favorable effect on clinical outcomes, including mortality risk [22,23,24,25,26]. However, the impact of immunomodulation treatments on the occurrence of thromboembolic events in COVID-19 patients remains uncertain, representing a clinically important gap in knowledge given the biologically plausible association between COVID-19-mediated inflammation and thrombosis.

Against this background, we performed a systematic review and meta-analysis of randomized controlled trials (RCTs) to investigate the effect of immunomodulatory agents on thromboembolic events in patients hospitalized with COVID-19.

## 2. Materials and Methods

### 2.1. Search Strategy and Inclusion Criteria

We searched PubMed and Scopus until 16 October 2021 for RCTs of immunomodulatory agents in COVID-19 reporting thromboembolic events. We used search items “hydroxychloroquine, corticosteroids, dexamethasone, hydrocortisone, prednisolone, interleukin-6 inhibitor, tocilizumab, sarilumab, siltuximab, interleukin-1 inhibitor, canakinumab, anakinra, complement inhibitor, vilobelimab, JAK-2 inhibitors, baricitinib, IVIG, TNFa inhibitor, interferon gamma, GM-CSF” and “coronavirus or COVID-19 or severe acute respiratory syndrome coronavirus 2 (SARS-CoV-2)” and “trial or randomized”. In addition, we searched the references of the related letters, reviews, and editorials to identify other potentially eligible studies. To be eligible for the analysis, the studies should have been RCTs, published as full-text articles in English, and provided data on venous and arterial thromboembolic events in patients hospitalized with COVID-19. RCTs of several other immunomodulatory agents not providing data on the incidence of thromboembolic events and pre-prints were not included in the analysis. This work was performed according to the PRISMA statement [27] and was submitted in PROSPERO (ID: CRD42021230346).

### 2.2. Outcomes, Data Extraction, and Assessment of the Risk of Bias

The following outcomes were assessed: venous thromboembolism (i.e., pulmonary embolism or deep vein thrombosis), myocardial infarction, ischemic stroke or systemic embolism, and the composite outcome of any thromboembolic event (TE) as reported in individual studies. Among the included studies, we assessed by means of a sensitivity analysis the outcome of mortality to explore potential correlations between thromboembolic events and mortality. In order to overcome the potential effect of hydroxychloroquine or corticosteroids which have been used as usual care in some studies, we prespecified one sensitivity analysis excluding hydroxychloroquine and corticosteroids treatment. Additionally, we assessed the outcome of pulmonary embolism among patients with VTE. Eligible studies were assessed independently by two authors (M.F. and P.T.), and the data were extracted using prespecified criteria and collection methods. An assessment of the risk of bias was performed by the same investigators with the use of the Cochrane Collaboration’s tool focusing on sequence generation, allocation concealment, blinding, addressing incomplete outcome data, selective reporting and presence of other bias. Any discrepancy or uncertainty was resolved by consensus or discussion among all authors.

### 2.3. Statistical Analysis

The data were analyzed on an intention-to-treat basis. Odds ratios (OR) and 95% confidence intervals (CI) were calculated for each outcome using the Mantel–Haenszel random effects method. Heterogeneity between trials was assessed by measuring inconsistency using the I^2^ index which measures the proportion of the variability in effect estimates that can be attributed to heterogeneity rather than chance. I^2^ was calculated as follows: I^2^ = 100% × (Q − df)/Q, where Q is the Cochran heterogeneity statistic and df is the degree of freedom. A value of 0% indicates no observed heterogeneity, and larger values show increasing heterogeneity [28]. The median follow-up duration was calculated using the follow-up duration reported in each trial.

We prespecified a subgroup analysis based on the immunomodulatory agent used in each study. Differences in pooled effect sizes between the subgroups were compared with a test of interaction (Cochran’s Q test).

All the analyses were performed with Review Manager 5 (RevMan) version 5.3 (Copenhagen, Denmark: The Nordic Cochrane Centre, The Cochrane Collaboration, 2011).

## 3. Results

The initial literature search yielded 1875 potentially eligible articles, of which 22 met the inclusion criteria [12,13,14,29,30,31,32,33,34,35,36,37,38,39,40,41,42,43,44,45,46,47]. The flow diagram of study selection is presented in Supplementary Figure S1. The main characteristics of the included trials are summarized in Table 1. Overall, we did not identify the major risk of bias, except in two studies where we identified high risk of bias in allocation concealment and blinding of participants [12,14] (Supplementary Figures S2 and S3).

Among 8499 patients hospitalized with COVID-19, 4635 were treated with an immunomodulatory agent as an add-on to usual care, and 3,861 were treated with usual care only. The median (IQR) follow-up period of the included studies was 28 (21–28) days.

### 3.1. Venous Thromboembolic Events

**Figure 1:**
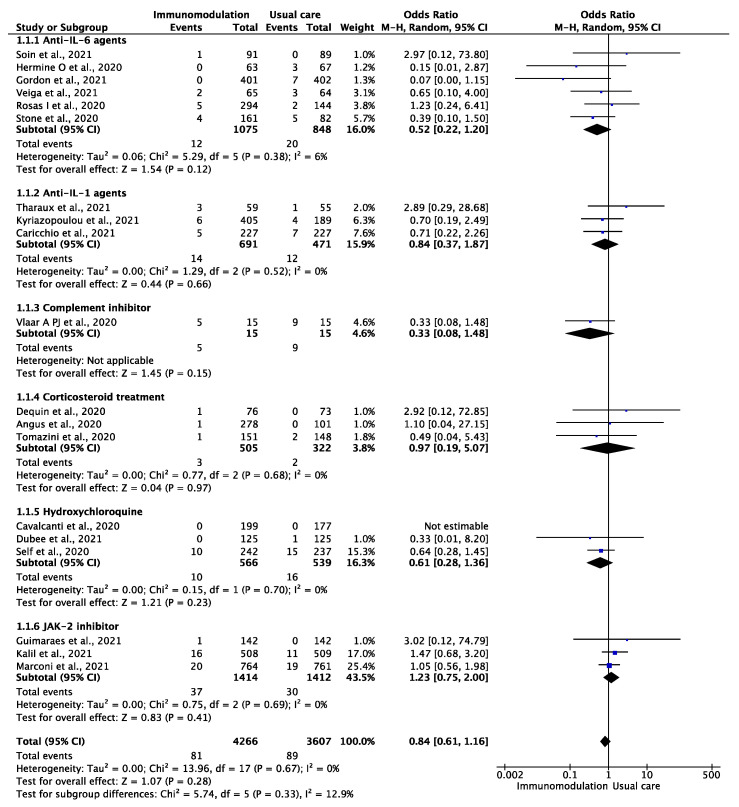
Odds ratio (OR) and 95% confidence intervals for the occurrence of venous thromboembolic events among the hospitalized COVID-19 patients prescribed immunomodulatory agents as an add-on to usual care vs. usual care only. Boxes represent the OR and lines represent the 95% CIs for individual studies. The diamonds and their width represent the pooled ORs and the 95% CIs, respectively. CI, confidence interval for the Mantel–Hansen estimator, I^2^: heterogeneity.

Among the 7873 patients from 18 trials [12,29,30,31,32,33,35,36,37,38,39,40,41,43,44,45,46,47] who were included in the analysis of the outcome of VTE comprising deep vein thrombosis and pulmonary embolism, during a median follow-up period of 28 (IQR: 21–28) days, the outcome occurred in 170 patients during the overall follow-up period of 8435 patient-months (2.01 per 100 patient-months). There were 81 VTE events among the COVID-19 patients prescribed an immunomodulatory agent (1.77 per 100 patient-months) and 88 among the placebo-assigned patients (2.30 per 100 patient-months) (OR: 0.84, 95% CI: 0.61–1.16; I^2^: 0%) (Figure 1). This effect remained consistent after excluding hydroxychloroquine and corticosteroid treatment (OR: 0.88, 95% CI: 0.61–1.27, I^2^: 3%). In the subgroup analysis of trials of IL-6 antagonists, there were 12 VTE events among the 1075 patients prescribed an IL-6 antagonist compared to 20 among the 848 patients in the placebo group (OR: 0.52, 95% CI: 0.22–1.20, RRR: 52%, ARR: 1.2%, NNT: 80) without heterogeneity (I^2^: 6%). In the sensitivity analysis of the outcome of pulmonary embolism, neither the overall immunomodulatory agents nor IL-6 antagonists significantly reduced the risk of pulmonary embolism (Supplementary Figure S4).

### 3.2. Ischemic Stroke or Systemic Embolism

**Figure 2:**
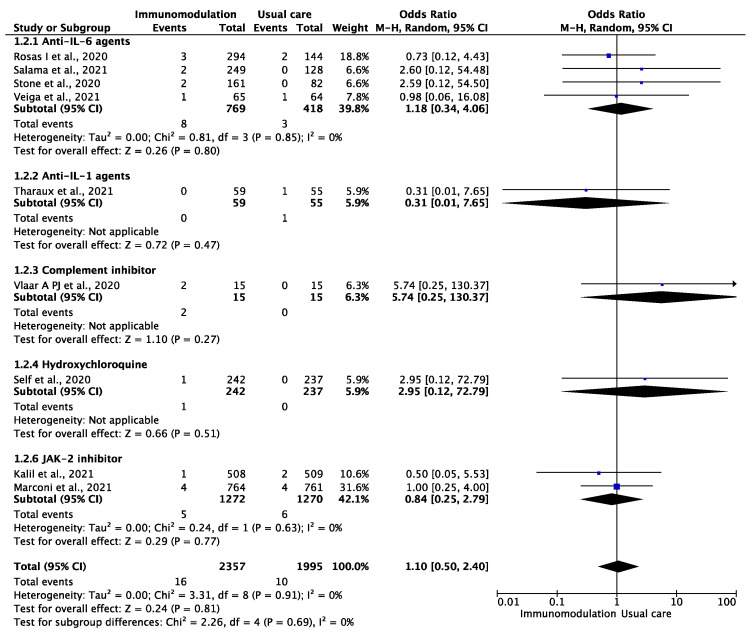
Odds ratio (OR) and 95% confidence intervals for the occurrence of ischemic stroke or systemic embolism among the hospitalized COVID-19 patients prescribed immunomodulatory agents as an add-on to usual care vs. usual care only. Boxes represent the OR and lines represent the 95% CIs for individual studies. The diamonds and their width represent the pooled ORs and the 95% CIs, respectively. CI, confidence interval for the Mantel–Hansen estimator, I2: heterogeneity.

Among the 4352 patients from nine trials [13,32,34,35,36,37,38,41,46] who were included in the analysis of ischemic stroke or systemic embolism, during a median follow-up period of 28 (IQR: 28–29) days, the outcome occurred in 26 patients during the follow-up period of 4663 patient-months (0.56 per 100 patient-months). There were 16 ischemic strokes or systemic embolic events among the patients prescribed immunomodulatory agents (0.63 per 100 patient-months) and 10 among the patients treated with SOC (0.46 per 100 patient-months) (OR: 1.10, 95% CI: 0.50–2.40; I^2^: 0%) (Figure 2). This effect remained consistent after excluding hydroxychloroquine treatment (OR: 1.03, 95% CI: 0.46–2.31, I^2^: 0%).

### 3.3. Myocardial Infarction

**Figure 3:**
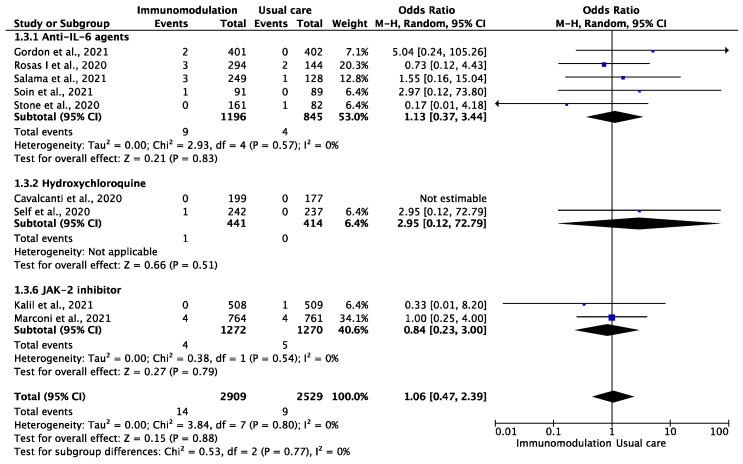
Odds ratio (OR) and 95% confidence intervals for the occurrence of myocardial infarction among the hospitalized COVID-19 patients prescribed immunomodulatory agents as an add-on to SOC vs. SOC only. Boxes represent the OR and lines represent the 95% CIs for individual studies. The diamonds and their width represent the pooled ORs and the 95% CIs, respectively. CI, confidence interval for the Mantel–Hansen estimator, I^2^: heterogeneity.

Among the 5438 patients from nine trials [13,31,32,33,34,35,39,40,46] who were included in the analysis of myocardial infarction, during a median follow-up period of 28 (IQR: 21–28) days, this outcome occurred in 23 patients during the overall follow-up period of 5826 patient-months (0.39 per 100 patient-months). There were 14 myocardial infarction events among the patients prescribed immunomodulatory agents (0.45 per 100 patient-months) and nine among the patients treated with usual care (0.33 per 100 patient-months) (OR: 1.06, 95% CI: 0.47–2.39; I^2^: 0%) (Figure 3). This effect remained consistent after excluding hydroxychloroquine treatment (OR: 0.99, 95% CI: 0.43–2.29, I^2^: 0%).

### 3.4. Any Thromboembolic Event and All-Cause Mortality

**Figure 4:**
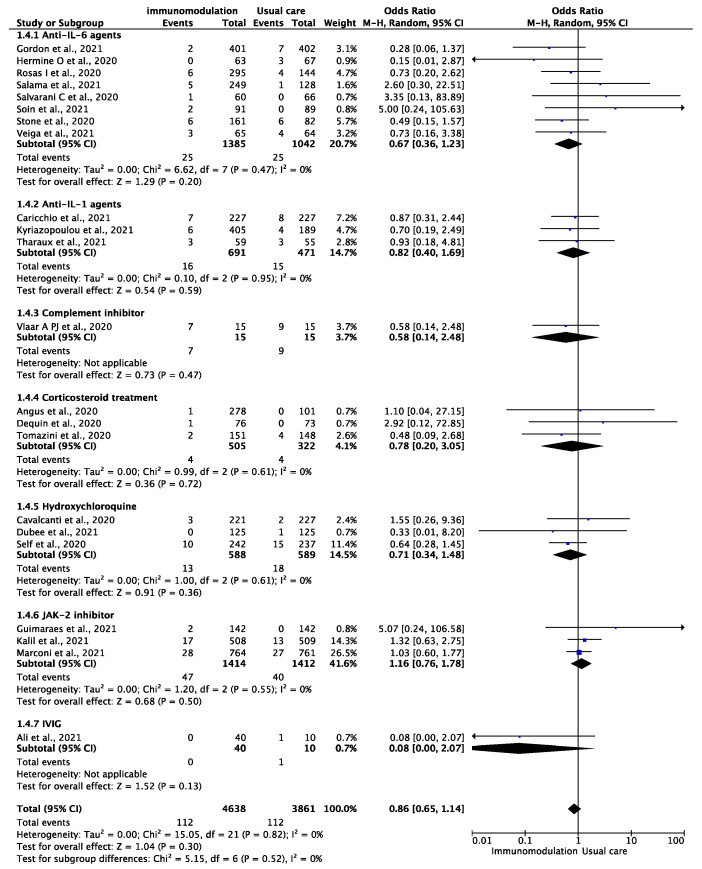
Odds ratio (OR) and 95% confidence intervals for the occurrence of any thromboembolic event among the hospitalized COVID-19 patients prescribed immunomodulatory agents as an add-on to SOC vs. SOC only. Boxes represent the OR and lines represent the 95% CIs for individual studies. The diamonds and their width represent the pooled ORs and the 95% CIs, respectively. CI, confidence interval for the Mantel–Hansen estimator, I^2^: heterogeneity.

Among the 8499 patients from 22 trials [12,13,14,29,30,31,32,33,34,35,36,37,38,39,40,41,42,43,44,45,46,47] who were included in the analysis of the composite outcome of any thromboembolic event, the outcome occurred in 224 patients during the follow-up period of 9106 patient-months (2.46 per 100 patient-months). There were 112 thromboembolic events among the patients prescribed immunomodulatory agents (2.25 per 100 patient-months) and 112 among the patients treated with the standard of care (2.71 per 100 patient-months) (OR: 0.86, 95% CI: 0.65–1.14; I^2^: 0%) (Figure 4). This effect remained consistent after excluding hydroxychloroquine and corticosteroid treatment (OR: 0.90, 95% CI: 0.66–1.22, I^2^: 0%). In the analysis of all-cause mortality, immunomodulation significantly reduced the risk of all-cause mortality in the included studies (OR: 0.76, 95% CI: 0.66–0.86, I^2^: 9%) (Supplementary Figure S5). In the sensitivity analysis excluding the effect of hydroxychloroquine and corticosteroid treatment, the results remained consistent (OR: 0.74, 95% CI: 0.64–0.87, I^2^: 18%).

## 4. Discussion

This meta-analysis did not identify a statistically significant effect of immunomodulation on the prevention of thromboembolic events in patients hospitalized with COVID-19. However, given the large effect estimate for the prevention of VTE, especially in patients treated with IL-6 antagonists, we cannot exclude an effect of immunomodulation on VTE occurrence. The effect of immunomodulation therapy on any thromboembolism, driven largely by VTE events, was a nonsignificant reduction in this outcome.

The potency of SARS-CoV-2 to trigger a proinflammatory cascade leading to a pulse of proinflammatory cytokines is the first step towards a vicious cycle of hyper-inflammation and cytokine release syndrome [6,7,8,9,10,48]. During this phase, TNF-α and IL-1β facilitate a sustained increase of IL-6. Furthermore, recent histopathological data suggest sustained systemic activation of the complement pathway in the microvascular network [49]. Moreover, locally activated platelets were shown to induce the release of neutrophil extracellular traps covered with the tissue factor, which in turn activates the extrinsic coagulation cascade leading to thrombin formation [50]. This cross-talk between innate immunity, platelets, and endothelial cells in the maladaptive host immune system can lead to the development of microvascular immune-mediated thrombosis and hypercoagulability [51]. Towards this direction, the effects of several immunomodulatory agents in COVID-19 are being investigated in ongoing randomized trials (Supplementary Table S1). In this context, it can be hypothesized that the administration of immunomodulatory agents such as IL-6 or IL-1 antagonists, complement inhibitors, corticosteroids, IVIG, and hydroxychloroquine acting on these pathogenic pathways could potentially mitigate the development of microvascular thrombosis with a corresponding reduction of VTE. Although our results did not confirm this hypothesis, we identified a trend towards fewer VTEs among patients treated with IL-6 inhibitors. Although this effect was not statistically significant, a potential effect of IL-6 antagonists on VTE risk cannot be excluded given the large effect estimate. We should not oversee that these results represent a potential additive effect of these agents on the antithrombotic effect of low-molecular-weight heparin, which was used in these studies. This was driven by RCTs of IL-6 inhibitors, but it should be noted that the number of events and patients in the IL-1-antagonist trials was low, and therefore a positive association cannot be excluded.

On the other hand, we did not identify any effect of these agents on arterial thromboembolic events comprising ischemic stroke, systemic embolism, and myocardial infarction. Compared to VTE, stroke is a heterogeneous syndrome comprising various pathophysiological mechanisms (e.g., atherosclerosis, atrial fibrillation, patent foramen ovale, dissection, small vessel disease), which frequently overlap [52,53,54,55,56]. This complexity may explain why immunomodulatory agents did not show any decrease in the risk of stroke or systemic embolism in the COVID-19 patients in our analysis.

Despite the potential myocardial injury which frequently occurs in patients with COVID-19 either due to myocardial infarction or because of inflammatory injury to the myocardial cells [57,58], we did not identify any effect of immunomodulatory agents on the risk of myocardial infarction. Although recently the RESCUE trial reported a marked reduction in inflammation and thrombosis biomarkers with a novel IL-6 inhibitor [59] and previous studies showed a beneficial effect of immunomodulatory agents in cardiovascular outcomes [60,61], this effect was not identified in our analysis of patients hospitalized with COVID-19. Currently, the effects of several immunomodulatory agents in COVID-19 are being investigated in ongoing randomized trials (Supplementary Table S1).

Strengths of this meta-analysis include the conduct and report of the analysis according to the PRISMA recommendations for reviews evaluating randomized trials [27]. In addition, our analysis did not consider observational studies, but focused only on RCTs characterized by their prospective design, which minimizes recall errors and selection bias, and the rigorous blind assessment of pre-defined and adjudicated outcome events, especially in this patient population, reduced the possible confounding effect of antithrombotic treatment.

There are potential limitations of this meta-analysis. Firstly, apart from the variations in the definitions of comorbidities used across trials, differences in patient selection criteria and differences in the length of follow-up across trials, the included immunomodulatory agents had highly distinctive immunological properties, which may have affected the outcomes of this study. Even though thromboembolic events and especially VTE were not systematically investigated in the included studies, most COVID-19-related thromboembolic events occur during the most severe, in-hospital period of the disease, and thus it is unlikely that these events were undocumented. Moreover, as these events were objectively diagnosed across treatment arms, no discrepancy would be likely to affect the observed treatment effects. Second, we acknowledge that the studies included in this meta-analysis were not designed to investigate the effect of immunomodulatory agents on thromboembolic events. As a result, the studies did not provide detailed data of thromboembolic events related to patient comorbidities and characteristics; thus, we were not able to perform further analyses, while the patients’ cardiovascular comorbidities may have affected the incidence of thromboembolic events in each arm. Additionally, the studies did not include extensive reports on antithrombotic treatment modalities used in these patients, and the outcomes were not independently adjudicated. However, since these events typically require objective diagnostic testing for confirmation and immunomodulation treatments are not known to have antithrombotic properties, it is unlikely that diagnostic suspicion bias may have affected detection of such events across the treatment arms. Lastly, as a significant number of clinical trials did not report results on thromboembolic events, some immunomodulatory agents were underrepresented in the analysis, potentially affecting the results of the study. Due to the increased research interest and publication rate related to COVID-19, additional RCTs may become available in the future and provide further insights in the effect of immunomodulation on the thromboembolic risk of COVID-19 patients.

## 5. Conclusions

In conclusion, we did not identify a statistically significant effect of immunomodulation on the prevention of thromboembolic events in patients hospitalized with COVID-19. However, given the large effect estimate for the prevention of VTE, especially in patients treated with IL-6 antagonists, we cannot exclude an effect of immunomodulation on VTE occurrence. The effect of immunomodulation on thromboembolic risk warrants further research.

## Figures and Tables

**Table 1:** Baseline characteristics of included studies.

Study	Drug	Included Patients	ICU	Male Gender	Age		Follow-Up (Days)	Standard of Care
					On Treatment	Control		
Stone et al., 2020 [35]	Tocilizumab	243	194	141	61.6 (46.4–69.7)	56.5 (44.7–67.8)	28	remdesivir
Vlaar A PJ et al., 2020 [38]	Vilobelimab	30	18	22	58	63	28	chloroquine, ganciclovir, azithromycin, nadroparin, LMWH, ASA, apixaban, rivaroxaban, clopidogrel, tinzaparin, dabigratan, edoxaban
Hermine O et al., 2020 [12]	Tocilizumab	130	0	88	64.0 (57.1–74.3)	63.3 (57.1–72.3)	28	antibiotics, antiviral agents, corticosteroids, vasopressor support, anticoagulants
Salvarani C et al., 2020 [14]	Tocilizumab	126	0	77	61.5 (51.5–73.5)	60 (54.0–69.0)	30	NA
Cavalcanti et al., 2020 [31]	HCQ	448	62	265	51.3 (36.8–65.8)	49.9 (34.8–65)	15	antibiotics, antiviral agents, corticosteroids
Dequin et al., 2020 [30]	Hydrocortisone	149	149	104	63.1 (51.5–70.8)	66.3 (53.5–72.7)	28	NA
Angus et al., 2020 [29]	Hydrocortisone	379	379	273	59.9 (47.7–72.1)	59.9 (45.3–74.5)	21	NA
Self et al., 2020 [32]	HCQ	479	96	267	58 (45–69)	57 (43–68)	28	NA
Tomazini et al., 2020 [40]	Dexamethasone	299	299	187	60.1 (15.8)	62.7 (13.1)	29	NA
Veiga et al., 2021 [36]	Tocilizumab	129	NA	88	57.4 (15.7)	57.5 (13.5)	29	HCQ, azithromycin, corticosteroids, antibiotics
Tharaux et al., 2021 [37]	Anakinra	114	NA	80	67 (55.5–74.3)	64.9 (59.5–78.3)	90	antibiotics, antiviral agents, corticosteroids, vasopressor support, anticoagulants
Salama et al., 2021 [13]	Tocilizumab	377	58	223	56 (14.3)	55.6 (14.9)	60	dexamethasone, remdesivir
Rosas I et al., 2021 [34]	Tocilizumab	438	0	306	60.9 (14.6)	60.6 (13.7)	60	remdesivir, glucocorticoids, convalescent plasma, supportive care
Gordon et al., 2021 [33]	Tocilizumab + Sarilumab	865	865	629	61.7 (12.7)	61.1 (12.7)	90	corticosteroids, remdesivir, COVID-19 IG, anticoagulants, macrolides, antiplatelet, statins
Soin et al., 2021 [39]	Tocilizumab	180	118	152	56 (47–63)	54 (43–63)	28	corticosteroids, remdesivir
Kalil et al., 2021 [41]	Baricitinib	1033	NA	652	55.4 (15.7)	55 (15.4)	29	corticosteroids, remdesivir
Ali et al., 2021 [42]	IVIG	50	NA	35	55.9 (1.34)	59.1 (12.1)	28	remdesivir, enoxaparin, antibiotic, dexamethasone/methylprednisolone
Caricchio et al., 2021 [44]	Canakinumab	454	0	267	59 (49–69)	57 (50–68)	29	heparin, dexamethasone, azithromycin, remdesivir, HCQ, convalescent plasma
Dubee et al., 2021 [43]	HCQ	250	0	121	76 (60–85)	78 (57–87)	28	azithromycin, other antibiotics, lopinavir-ritonavir, corticosteroids
Kyriazopoulou et al., 2021 [45]	Anakinra	594	42	344	62 (11.4)	61.5 (11.3)	28	dexamethasone, LMWH, remdesivir, antibiotics
Marconi et al., 2021 [46]	Baricitinib	1525	0	963	57.8 (14.3)	57.6 (13.8)	28	dexamethasone, remdesivir
Guimaraes et al., 2021 [47]	Tofacitinib	289	54	188	55 (14)	57 (14)	28	glucocorticosterotds, antibiotics, remdesivir

HCQ, Hydroxycloroquinw; NA, not applicable; IVIG, intravenous immunoglobulin; LMWH, low molecular-weight heparin; ASA, acetylsalicylic acid.

## Data Availability

Data sharing not applicable. No new data were created or analyzed in this study. Data sharing is not applicable to this article.

## Supplementary Materials

The following are available online at https://www.mdpi.com/article/10.3390/jcm10225366/s1, Figure S1: Flow diagram of the studies identified, screened, and included in the meta-analysis, Figure S2: (A) Risk of bias graph: review of the authors’ judgements about each risk of bias item presented as percentages across all the included studies; (B) risk of bias summary: review of the authors’ judgements about each risk of bias item for each included study, Figure S3: Diagnostic plots for each outcome based on the use of immunomodulatory agents or placebo. Panel (A): venous thromboembolism, Panel (B): ischemic stroke or systemic embolism, Panel (C): myocardial infarction, Panel (D): Any thromboembolic event, Figure S4: Odds ratio (OR) and 95% confidence intervals for the occurrence of pulmonary embolism among the hospitalized COVID-19 patients prescribed immunomodulatory agents as an add-on to SOC vs. SOC only. Boxes represent the OR and lines represent the 95% CIs for individual studies. The diamonds and their width represent the pooled ORs and the 95% CIs, respectively. CI, confidence interval for the Mantel–Hansen estimator, I^2^: heterogeneity, Figure S5: Odds ratio (OR) and 95% confidence intervals for the occurrence of all-cause mortality in the included studies among the hospitalized COVID-19 patients prescribed immunomodulatory agents as an add-on to SOC vs. SOC only. Boxes represent the OR and lines represent the 95% CIs for individual studies. The diamonds and their width represent the pooled ORs and the 95% CIs, respectively. CI, confidence interval for the Mantel–Hansen estimator, I^2^: heterogeneity; Table S1: Studies of immunomodulatory agents for COVID-19.
