# Supplementary material for: Immunomodulation and Reduction of Thromboembolic Risk in Hospitalized COVID-19 Patients: Systematic Review and Meta-Analysis of Randomized Trials
Source: J Clin Med. 2021 Nov 18;10(22):5366. doi: 10.3390/jcm10225366 (PMC8617689; doi:10.3390/jcm10225366)
Supplement: Supplementary file 1 [file jcm-10-05366-s001.zip › jcm-1447520-supplementary.pdf]

Supplemental material

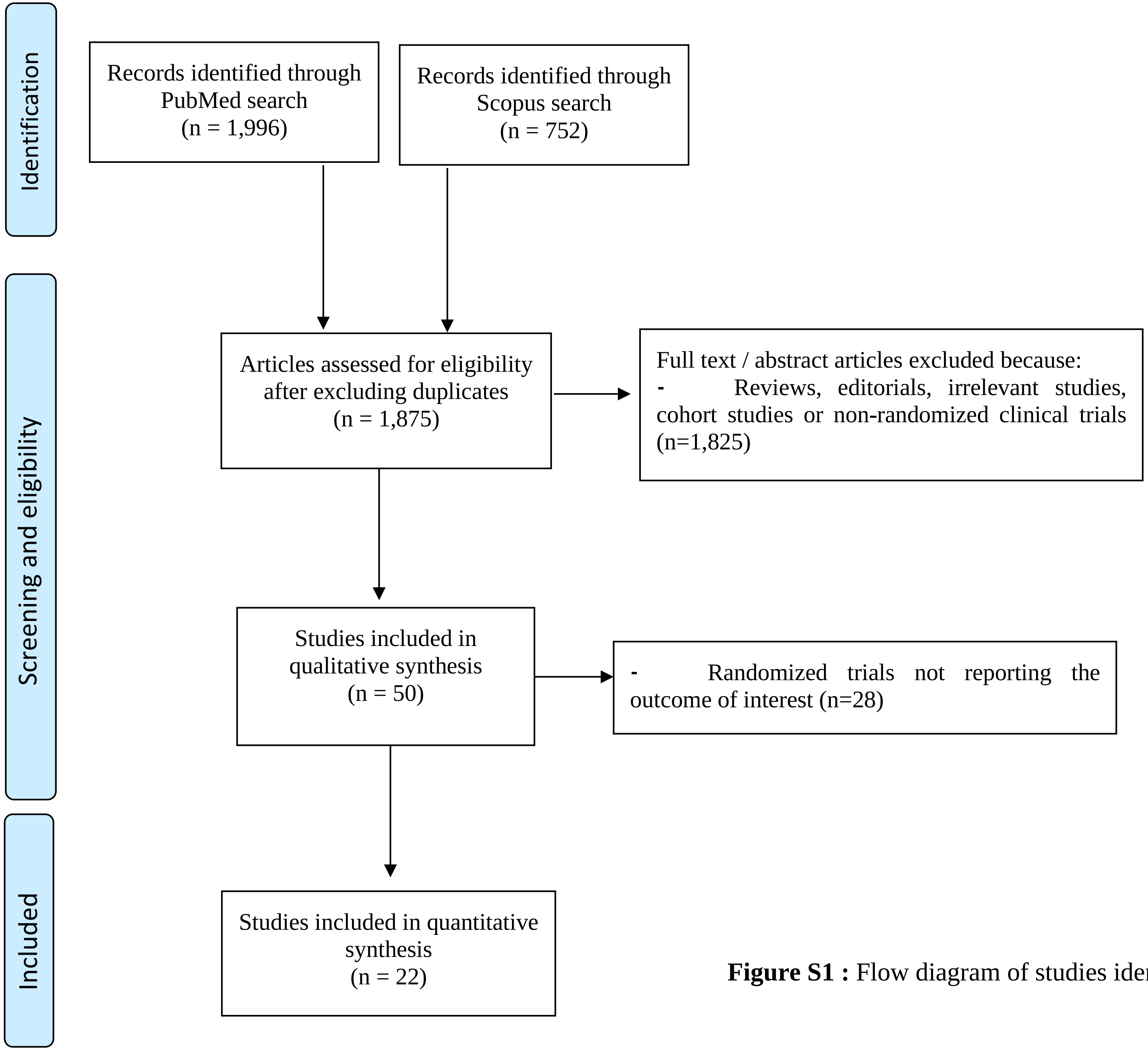

Figure S1 : Flow diagram of studies identified, screened and included in the meta-analysis

Table S1. Studies of immunomodulatory agents for COVID-19

Drug	Drug class	Number of clinical trials (completed or ongoing)	Available studies
**tocilizumab**	anti-IL6	72	Clinical trials
**adalimumab**	anti-TNFa	1	Clinical trial
**infliximab**	anti-TNFa	5	Clinical trials
**baricitinib**	Jak inhibitor	18	Clinical trials
**ruxolitinib**	Jak inhibitor	16	Clinical trials
**interferon**	Signaling protein - Immunomodulatory agent	141	Clinical trials
**IVIG**	Immunomodulatory agent	23	Clinical trials
**colchicine**	Anti-inflammatory		Clinical trials
**GM-CSF (e.g. sargramostin)**	GM-CSF	41	Clinical trials
**anakinra**	anti-IL1	31	Clinical trials
**hyperimmune or convalescent plasma**	-	460	Clinical trials

GM-CSF: Granulocyte-macrophage colony-stimulating factor, IL: interleukin, IVIG: intravenous immunoglobulin, Jak: Janus kinase, TNF: tumor necrosis factor

B.

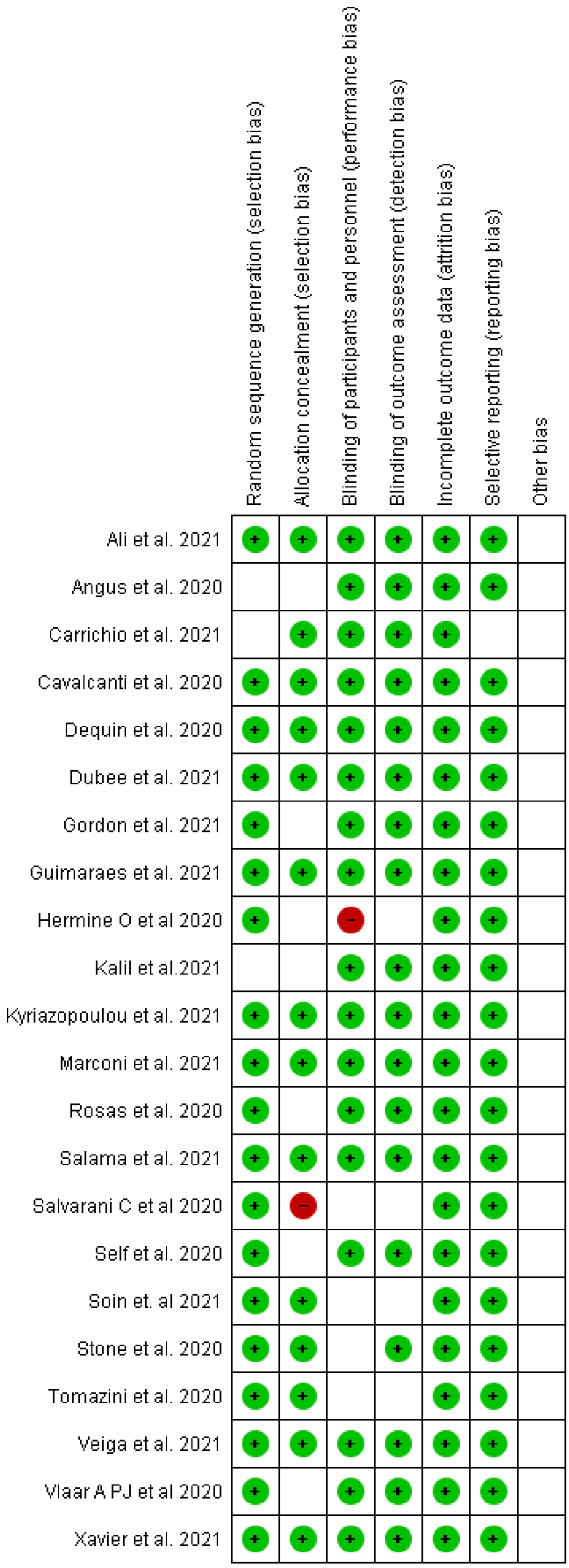

A.

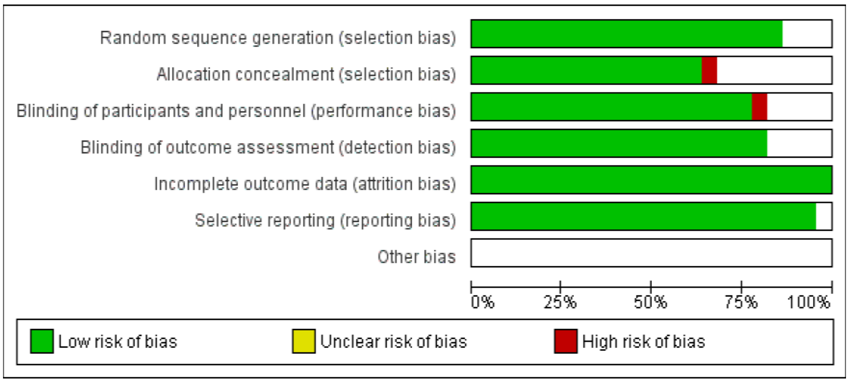

Figure S2.A. Risk of bias graph: review authors' judgements about each risk of bias item presented as percentages across all included studies; **B.**Risk of bias summary: review authors' judgements about each risk of bias item for each included study.

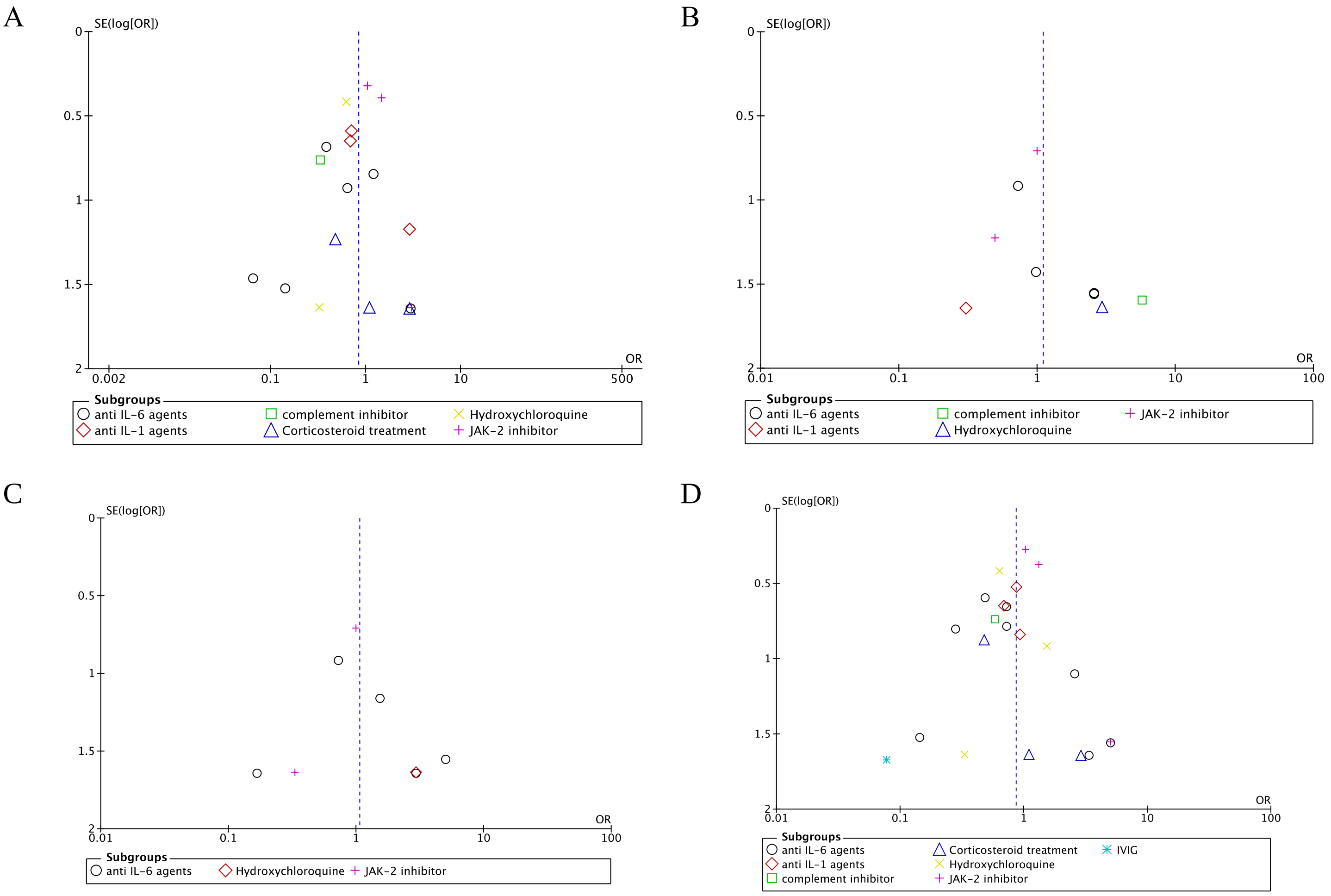

Figure S3 . Diagnostic plots for each outcome based on the use of immunomodulatory agents or placebo.Panel A: Venous thromboembolism, Panel B: Ischemic Stroke or systemic embolism, Panel C: myocardial infarction, Panel D: Any thromboembolic event

Study or Subgroup	Experimental Events	Experimental Total	Control Events	Control Total	Weight	Odds Ratio M-H, Fixed, 95% CI
**1.5.1 anti IL-6 agents**						
Hermine O et al 2020	0	63	3	67	7.6%	0.15 [0.01, 2.87]
Soin et al. 2021	1	91	0	89	1.1%	2.97 [0.12, 73.80]
Stone et al. 2020	2	161	2	82	5.9%	0.50 [0.07, 3.64]
Veiga et al 2021	3	65	0	64	1.1%	7.22 [0.37, 142.73]
**Subtotal (95% CI)**		**380**		**302**	**15.7%**	**0.97 [0.34, 2.79]**
Total events	6		5			
Heterogeneity: Chi^2 = 4.18, df = 3 (P = 0.24); I^2 = 28%						
Test for overall effect: Z = 0.06 (P = 0.95)						
**1.5.2 anti IL-1 agents**						
Caricchio et al.2021	3	227	5	227	11.1%	0.59 [0.14, 2.52]
Kyriazopoulou et al. 2021	6	405	4	189	12.1%	0.70 [0.19, 2.49]
Xavier et al 2021	3	59	1	55	2.2%	2.89 [0.29, 28.68]
**Subtotal (95% CI)**		**691**		**471**	**25.5%**	**0.84 [0.36, 1.98]**
Total events	12		10			
Heterogeneity: Chi^2 = 1.42, df = 2 (P = 0.49); I^2 = 0%						
Test for overall effect: Z = 0.39 (P = 0.70)						
**1.5.3 complement inhibitor**						
Vlaar A PJ et al 2020	5	15	9	15	13.5%	0.33 [0.08, 1.48]
**Subtotal (95% CI)**		**15**		**15**	**13.5%**	**0.33 [0.08, 1.48]**
Total events	5		9			
Heterogeneity: Not applicable						
Test for overall effect: Z = 1.45 (P = 0.15)						
**1.5.4 Corticosteroid treatment**						
Angus et al.	1	278	0	101	1.6%	1.10 [0.04, 27.15]
Dequin et al.	1	76	0	73	1.1%	2.92 [0.12, 72.85]
Tomazini et al 2020	0	151	1	148	3.4%	0.32 [0.01, 8.03]
**Subtotal (95% CI)**		**505**		**322**	**6.2%**	**1.00 [0.19, 5.35]**
Total events	2		1			
Heterogeneity: Chi^2 = 0.90, df = 2 (P = 0.64); I^2 = 0%						
Test for overall effect: Z = 0.00 (P = 1.00)						
**1.5.5 Hydroxychloroquine**						
Dubee et al.2021	0	125	1	125	3.4%	0.33 [0.01, 8.20]
Self et al, 2020	4	242	5	237	11.2%	0.78 [0.21, 2.94]
**Subtotal (95% CI)**		**367**		**362**	**14.6%**	**0.68 [0.20, 2.27]**
Total events	4		6			
Heterogeneity: Chi^2 = 0.24, df = 1 (P = 0.63); I^2 = 0%						
Test for overall effect: Z = 0.63 (P = 0.53)						
**1.5.6 JAK-2 inhibitor**						
Kalil et al, 2021	5	508	2	509	4.5%	2.52 [0.49, 13.05]
Marconi et al. 2021	13	764	9	761	20.0%	1.45 [0.61, 3.40]
**Subtotal (95% CI)**		**1272**		**1270**	**24.5%**	**1.64 [0.77, 3.49]**
Total events	18		11			
Heterogeneity: Chi^2 = 0.34, df = 1 (P = 0.56); I^2 = 0%						
Test for overall effect: Z = 1.29 (P = 0.20)						
**Total (95% CI)**		**3230**		**2742**	**100.0%**	**0.97 [0.64, 1.48]**
Total events	47		42			
Heterogeneity: Chi^2 = 11.31, df = 14 (P = 0.66); I^2 = 0%						
Test for overall effect: Z = 0.12 (P = 0.90)						
Test for subgroup differences: Chi^2 = 4.29, df = 5 (P = 0.51), I^2 = 0%						

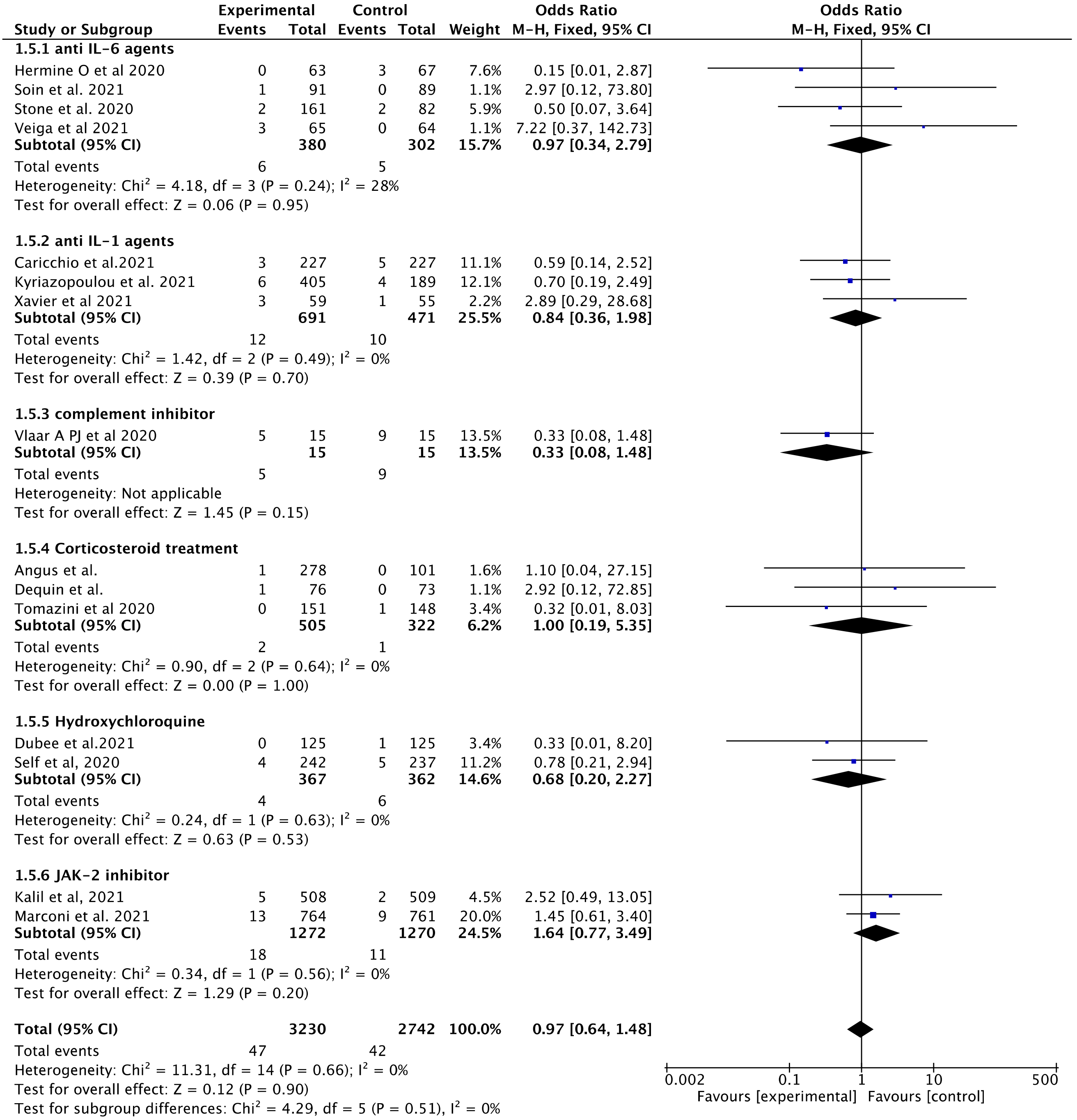

Figure S4: Odds ratio (OR) and 95% Confidence Intervals for the occurrence of pulmonary embolism among hospitalized COVID-19 patients assigned to immunomodulatory agents as an add-on to SOC vs. SOC only. Boxes represent the OR and lines represent the 95% CIs for individual studies. The diamonds and their width represent the pooled ORs and the 95% CIs, respectively. CI, confidence intervals for the Mantel-Hansen estimator, I^2: heterogeneity

Study or Subgroup	Experimental Events	Experimental Total	Control Events	Control Total	Weight	Odds Ratio M-H, Fixed, 95% CI
**1.6.1 anti IL-6 agents**						
Gordon et al.	108	395	142	397	20.0%	0.68 [0.50, 0.91]
Hermine O et al 2020	7	63	8	67	1.3%	0.92 [0.31, 2.71]
Rosas I et al 2020	58	295	28	144	5.9%	1.01 [0.61, 1.68]
Salama et al	26	249	11	128	2.5%	1.24 [0.59, 2.60]
Salvarani C et al 2020	2	60	1	66	0.2%	2.24 [0.20, 25.37]
Soin et al. 2021	13	91	15	89	2.5%	0.82 [0.37, 1.84]
Stone et al. 2020	9	161	4	82	1.0%	1.15 [0.34, 3.87]
Veiga et al 2021	14	65	6	64	0.9%	2.65 [0.95, 7.42]
**Subtotal (95% CI)**		**1379**		**1037**	**34.3%**	**0.87 [0.70, 1.08]**
Total events	237		215			
Heterogeneity: Chi^2 = 9.28, df = 7 (P = 0.23); I^2 = 25%						
Test for overall effect: Z = 1.26 (P = 0.21)						
**1.6.2 anti IL-1 agents**						
Caricchio et al.2021	25	227	32	227	5.5%	0.75 [0.43, 1.32]
Kyriazopoulou et al. 2021	13	405	13	189	3.3%	0.45 [0.20, 0.99]
Xavier et al 2021	16	59	16	55	2.3%	0.91 [0.40, 2.05]
**Subtotal (95% CI)**		**691**		**471**	**11.2%**	**0.70 [0.47, 1.04]**
Total events	54		61			
Heterogeneity: Chi^2 = 1.67, df = 2 (P = 0.43); I^2 = 0%						
Test for overall effect: Z = 1.79 (P = 0.07)						
**1.6.3 complement inhibitor**						
Vlaar A PJ et al 2020	2	15	4	15	0.7%	0.42 [0.06, 2.77]
**Subtotal (95% CI)**		**15**		**15**	**0.7%**	**0.42 [0.06, 2.77]**
Total events	2		4			
Heterogeneity: Not applicable						
Test for overall effect: Z = 0.90 (P = 0.37)						
**1.6.4 Corticosteroid treatment**						
Angus et al.	78	278	33	99	6.8%	0.78 [0.48, 1.28]
Dequin et al.	11	76	20	73	3.4%	0.45 [0.20, 1.02]
Tomazini et al 2020	85	151	91	148	7.8%	0.81 [0.51, 1.28]
**Subtotal (95% CI)**		**505**		**320**	**18.0%**	**0.73 [0.53, 0.99]**
Total events	174		144			
Heterogeneity: Chi^2 = 1.60, df = 2 (P = 0.45); I^2 = 0%						
Test for overall effect: Z = 2.00 (P = 0.05)						
**1.6.5 Hydroxychloroquine**						
Cavalcanti et al	8	221	5	227	0.9%	1.67 [0.54, 5.18]
Dubee et al.2021	6	125	11	125	2.0%	0.52 [0.19, 1.46]
Self et al, 2020	25	242	25	237	4.4%	0.98 [0.54, 1.76]
**Subtotal (95% CI)**		**588**		**589**	**7.4%**	**0.94 [0.59, 1.48]**
Total events	39		41			
Heterogeneity: Chi^2 = 2.26, df = 2 (P = 0.32); I^2 = 11%						
Test for overall effect: Z = 0.27 (P = 0.78)						
**1.6.6 JAK-2 inhibitor**						
Kalil et al, 2021	24	508	37	509	6.8%	0.63 [0.37, 1.07]
Marconi et al. 2021	79	764	116	761	20.2%	0.64 [0.47, 0.87]
**Subtotal (95% CI)**		**1272**		**1270**	**27.1%**	**0.64 [0.49, 0.83]**
Total events	103		153			
Heterogeneity: Chi^2 = 0.00, df = 1 (P = 0.97); I^2 = 0%						
Test for overall effect: Z = 3.32 (P = 0.0009)						
**1.6.7 IVIG**						
Ali et al. 2021	10	40	6	10	1.4%	0.22 [0.05, 0.95]
**Subtotal (95% CI)**		**40**		**10**	**1.4%**	**0.22 [0.05, 0.95]**
Total events	10		6			
Heterogeneity: Not applicable						
Test for overall effect: Z = 2.03 (P = 0.04)						
**Total (95% CI)**		**4490**		**3712**	**100.0%**	**0.76 [0.66, 0.86]**
Total events	619		624			
Heterogeneity: Chi^2 = 21.97, df = 20 (P = 0.34); I^2 = 9%						
Test for overall effect: Z = 4.20 (P < 0.0001)						
Test for subgroup differences: Chi^2 = 7.36, df = 6 (P = 0.29), I^2 = 18.5%						

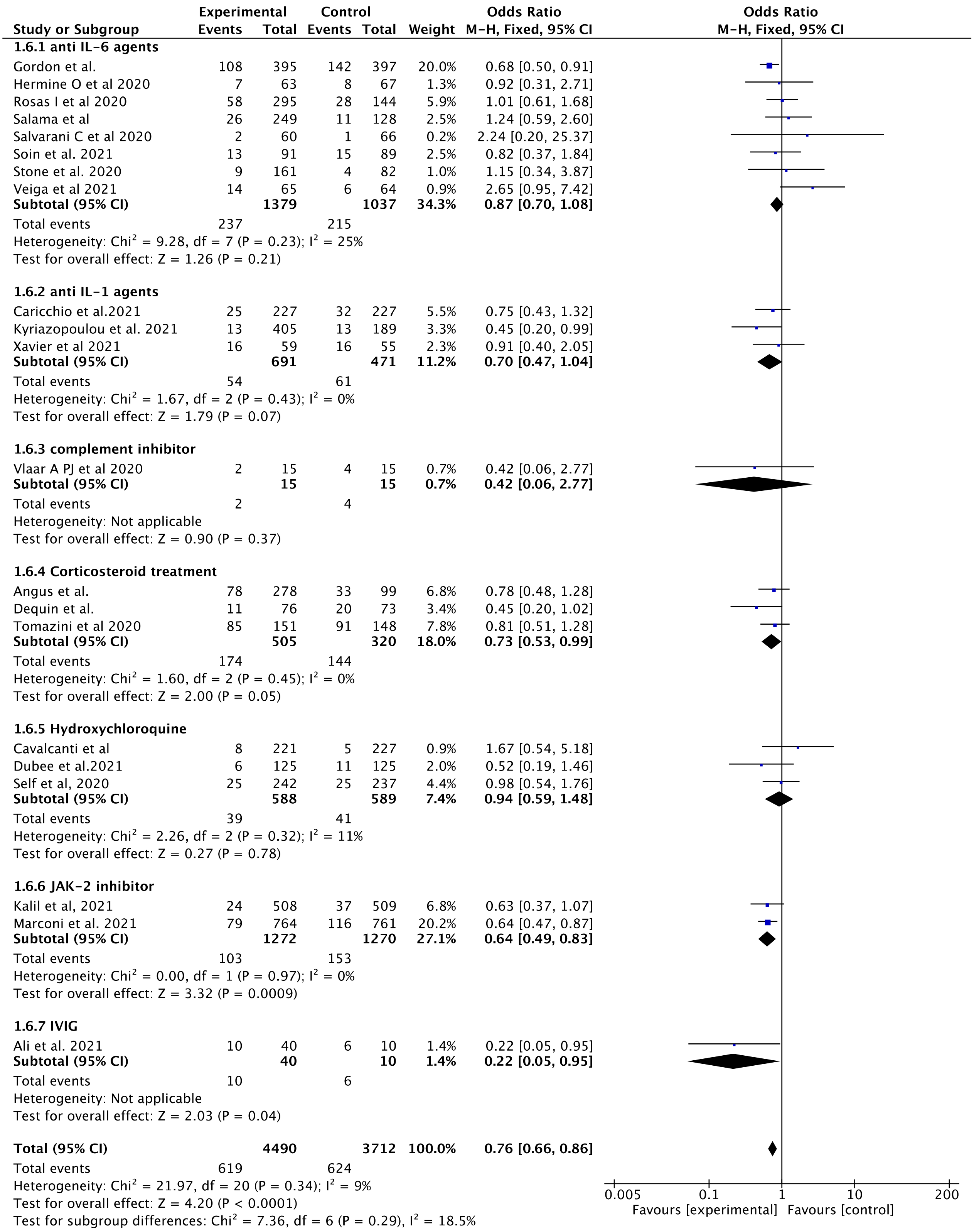

Figure S5: Odds ratio (OR) and 95% Confidence Intervals for mortality among hospitalized COVID-19 patients assigned to immunomodulatory agents as an add-on to SOC vs. SOC only. Boxes represent the OR and lines represent the 95% CIs for individual studies. The diamonds and their width represent the pooled ORs and the 95% CIs, respectively. CI, confidence intervals for the Mantel-Hansen estimator, I^2: heterogeneity
